# Fatty acid composition of goose meat depending on genotype and sex

**DOI:** 10.5713/ajas.17.0672

**Published:** 2018-04-12

**Authors:** Linda Uhlířová, Eva Tůmová, Darina Chodová, Zdeněk Volek, Vlastislav Machander

**Affiliations:** 1 Department of Animal Husbandry, Czech University of Life Sciences Prague, Kamýcká 129, 165 21 Prague – Suchdol, Czech Republic; 2 Department of Physiology of Nutrition and Product Quality, Institute of Animal Science, Přátelství 815, 104 00 Prague – Uhříněves, Czech Republic; 3 International Poultry Testing, Ústrašice, 390 02 Tábor, Czech Republic

**Keywords:** Genotype, Fatty Acid Composition, Sex, Lipid Metabolism, Oxidative Stability

## Abstract

**Objective:**

The aim of this study was to compare male and female geese of two contrasting genotypes in terms of fatty acid composition, indexes related to human health, lipid metabolism and oxidative stability of the meat.

**Methods:**

The experiment was carried out on total of 120 geese of two different genotypes; the native breed Czech goose (CG) and commercial hybrid Novohradska goose (NG). One-d-old goslings were divided into 4 groups according to genotype and sex, and 8 birds from each group were slaughtered at 8 weeks of age.

**Results:**

The effects of the interactions between genotype and sex were observed on growth performance and carcass traits. Final body weight (p<0.001), daily weight gain (p<0.001), daily feed intake (p<0.001), slaughter weight (p<0.001), and cold carcass weight (p<0.001) were highest in NG males and lowest in CG females. The meat fatty acid composition results showed effects of both genotype and sex on the total n-6 and the total polyunsaturated fatty acid (PUFA) content, as well as the PUFA n-6/PUFA n-3 ratio. Regarding genotype, the total n-6, the total PUFA content and the PUFA n-6/PUFA n-3 ratio were higher in CG, and higher values were found in females. In terms of the lipid metabolism, Δ^5^–Δ^6^ desaturase (p = 0.006) was higher in males. The meat oxidative stability results revealed an interaction between genotype, sex and storage time (p<0.001). The highest (13.85 mg/kg) malondialdehyde content was measured in the meat of CG females after 5 days of storage and was presumably related to a higher PUFA content.

**Conclusion:**

NG had a relatively higher growth rate and meat oxidative stability, whereas the advantage of CG meat is its favourable fatty acid profile characterized by a higher PUFA content.

## INTRODUCTION

Consumer interest in meat from sustainable farming continues to grow [1]. Local, slow-growing (native) breeds of poultry are recommended for extensive rearing because while they do not perform as well as hybrid birds, they are characterized by very good health, resistance to adverse climatic conditions [2], and high-quality meat [3].

Meat quality, including the fatty acid profile, is influenced by both genetic and environmental factors [4], but, genetic factors have less of an influence on meat fatty acid composition than environmental, especially dietary, ones. Moreover, differences in the fatty acid profile between genotypes are related to differences in the fat content of the meat [5] and the attention paid to the fatty acid profile of meat mainly stems from consumer demand for healthier food [6]. Geese fat is generally considered to be relatively safe in terms of consumer health due to its high content of oleic, linoleic, linolenic and arachidonic acids, all of which are products of the enzymatic desaturation of stearic acid [7].

Beyond the significant influence of meat fatty acid composition on human health, fatty acids are involved in various technological aspects of meat quality [6] such as lipid oxidation, which leads to discolouration, drip losses, off-odour and off-flavour development, and the production of potentially toxic compounds. Low oxidative stability has a negative impact on further meat storage and processing [8].

Several studies of the effect of genotype on the meat fatty acid profile of geese have been conducted, and they compared different native Polish geese breeds [7,2], two strains of White Kołuda geese [9], and a pedigree strain of the Chinese Yangzhou goose with its 4 crossbreeds [10]. However, there have been no studies comparing a native goose breed with a hybrid genotype in terms of the fatty acid profile and lipid metabolism of the meat. Moreover, information about the oxidative stability of goose meat is limited. Previous experiments [11,12] have dealt with the effect of nutrition on meat lipid oxidation, but the effect of genotype has not yet been described. The aim of the present study was to compare male and female geese of two contrasting genotypes in terms of the fatty acid composition, indexes related to human health, lipid metabolism and the oxidative stability of the meat.

## MATERIALS AND METHODS

The experiment was conducted at International Poultry Testing in Ústrašice and approved by the Ethics Committee of the Czech University of Life Sciences Prague and the Central Commission for Animal Welfare at the Ministry of Agriculture of the Czech Republic.

### Animals and experimental design

The experiment was carried out on 20 males and 20 females of the native breed Czech goose (CG) and 40 males and 40 males of the crossbreed Novohradska goose (NG), i.e. a total of 120 birds. After being weighed one-d-old goslings were divided into 4 groups according to genotype and sex and housed in pens on litter (20 goslings per pen; 6 birds per m^2^). The daily photoperiod was as follows: 24 h of light for the first three days of the fattening period, then 16 h of light and 8 h of darkness between the 4th and 7th days of the fattening period, and then 14 h of light and 10 h of darkness from the 8th day. The room temperature was 32°C in the 1st wk of fattening period, then gradually decreased by aproximatelly 2°C every 3 days so that at 4 wk of age of goslings the temperature was 18°C to 20°C, which was maintained until the end of fattening. Relative humidity was 50% to 65%. During the experiment, two types of feed mixtures were provided (Table 1): feed mixture VH 1 until the goslings were 4 wk of age and VH 2 until the end of the experiment, which ran until the goslings were 8 wk of age. Feed mixtures as well as drinking water were offered *ad libitum*, and the body weight of the goslings and their feed consumption were recorded weekly.

A sample of the birds was slaughtered at the end of the experiment (when the goslings reached 8 wk of age). Eight birds of average body weight from each group (32 in total) were stunned, slaughtered by cutting their carotid arteries and then immediately bled. The heads and feet were removed, and the carcasses were then eviscerated and, after 24 h of chilling at 4°C, weighed to obtain the cold carcass weight. The abdominal fat and thighs were removed and weighed, and the thighs were then deboned to obtain the weight of the thigh meat. The dressing-out percentage was calculated as the percentage of the cold carcass weight relative to the slaughter weight, and the thighs, thigh meat and abdominal fat percentages were calculated relative to the cold carcass weight. Samples of the thigh meat were stored at −40°C until analysis.

### Analytical determinations

The meat dry matter was determined by drying in an oven at 105°C, and the free fat content was obtained by extraction with petroleum ether in a Soxtec 1043 apparatus (FOSS Tecator AB, Höganäs, Sweden). The free fat was determined according to ISO 1444 [13].

The fatty acid composition of the thigh meat was determined following chloroform-methanol extraction of the total lipids in accordance with Folch et al [14], which consisted of homogenizing of the tissue with a 2:1 chloroform-methanol mixture and washing the extract by addition of 0.2 its volume of water. The resulting mixture separated into two phases, the lower phase was the the total pure lipid extract. Nonadecanoic acid was used as an internal marker to quantify the fatty acids in the samples. Alkaline transmethylation of the fatty acids was performed according to Raes et al [15]. The isolated methyl esters contents were determined using an HP 6890 gas chromatograph (Agilent Technologies, Inc., Santa Clara, CA, USA) equipped with a flame-ionisation detector and a programmed 60 m DB-23 capillary column (150°C to 230°C), and split injections were performed using an Agilent autosampler. Fatty acids were identified based on retention times relative to the following standards: PUFA No. 1 Mix, PUFA No. 2 Mix, PUFA No. 3 Mix and 37 Component FAME Mix (Supelco, Bellefonte, PA, USA). All analyses were performed in duplicate.

The oxidative stability of the thigh meat was determined by the thiobarbituric acid method according to Piette and Raymond [16], which is based on spectrophotometric measurement of the color intensity of the products derived from the reaction of thiobarbituric acid with the meat oxidation products. Measurements were performed after 0, 3, and 5 days of meat storage at 4°C, and the thiobarbituric acid reactive substances (TBARS) were expressed as mg of malondialdehyde (MDA) per kg of meat.

### Calculations and statistical analysis

The atherogenicity index (AI) and thrombogenicity index (TI) were calculated according to Ulbricht and Southgate [17]; the peroxidability index (PI) was calculated following Arakawa and Sagai [18]; and the hypocholesterolemic/hypercholesterolemic ratio (HH) was calculated in accordance with Santos-Silva et al [19]. The equations are listed below.

$$\begin{array}{l} \text{AI}=(\text{C}12:0+4\times \text{C}14:0+\text{C}16:0)/(\Sigma \text{MUFA}+\Sigma \text{n-}6+\Sigma \text{n-}3)\\ \text{TI}=(\text{C}14:0+\text{C}16:0+\text{C}18:0)/[(0.5\times \Sigma \text{MUFA})+(0.5\times \Sigma \text{n-}6)+(3\times \Sigma \text{n-}3)+(\Sigma \text{n-}3/\Sigma \text{n-}6)]\\ \text{PI}=(\%\;\text{monoenoic}\times 0.025)+(\%\;\text{dienoic}\times 1)\\ +(\%\;\text{trienoic}\times 2)+(\%\;\text{tetraenoic}\times 4)\\ +(\%\;\text{pentaenoic}\times 6)+\%\;\text{hexaenoic}\times 8)\\ \text{HH}=(\text{C}18:1\;\text{n-}9+18:2\text{n-}6+18:3\text{n-}3+20:5\text{n-}3+22:6\text{n-}3)/(\text{C}14:0+16:0) \end{array}$$

The lipid metabolism indexes were calculated using the following equations from Dal Bosco et al [4].

$$\begin{array}{l} \text{Elongase}=\text{C}18:0/\text{C}16:0\\ \text{Thioesterase}=\text{C}16:0/\text{C}14:0\\ \Delta^{9}\;\text{desaturase }(16)=100\times [\text{C}16:1\text{n-}9/(\text{C}16:1\text{n-}9+\text{C}16:0)]\\ \Delta^{9}\;\text{desaturase }(18)=100\times [\text{C}18:1\text{n-}9/(\text{C}18:1\text{n-}9+\text{C}18:0)]\\ \Delta^{9}\;\text{desaturase }(16+18)=100\times [(\text{C}16:1\text{n-}9+\text{C}18:1\text{n-}9)/(\text{C}16:1\text{n-}9+\text{C}16:0+\text{C}18:1\text{n-}9+\text{C}18:0)]\\ \Delta^{5}=\Delta^{6}\;\text{desaturase}\\ =100\times [(\text{C}20:2\text{n-}6+\text{C}20:4\text{n-}6+\text{C}20:5\text{n-}3+\text{C}22:5\text{n-}3+\text{C}22:6\text{n-}3)/\\ (\text{C}18:2\text{n-}6+\text{C}18:3\text{n-}3+\text{C}20:2\text{n-}6+\text{C}20:4\text{n-}6+\\ \text{C}20:5\text{n-}3+\text{C}22:5\text{n-}3+\text{C}22:6\text{n-}3)] \end{array}$$

Statistical analysis was performed using the general linear model procedure of SAS software [20]. The growth performance and carcass traits data as well as chemical composition, fatty acid composition, nutritional indexes and lipid metabolism indexes of the goose meat were analysed by two-way analysis of variance including the interaction of genotype and sex, whereas the oxidative stability of the goose meat was analysed by multiple analysis of variance with the interaction of genotype, sex and storage time. Differences between means were tested by Scheffé’s test and considered as statistically significant at p<0.05.

## RESULTS

### Growth performance and carcass traits

The growth performance results are shown in Table 2. A highly significant effect of genotype was detected in all the studied characteristics. A higher initial body weight (by 16.7%; p<0.001), final body weight (by 24.3%; p<0.001), daily weight gain (by 24.3%; p<0.001) and daily feed intake (by 16.7%; p<0.001) were found in NG, whereas feed conversion was higher (by 5.4%; p<0.001) in CG. An effect of sex was found in both the initial (p<0.001) and final body weights (p<0.001), daily weight gain (p<0.001) and daily feed intake (p<0.001). The initial weight was higher in females (by 4.1%; p<0.001), but after 8 weeks the final body weight was higher (by 13.4%; p<0.001) in males. Interactions between genotype and sex were recorded in all the studied characteristics.

The carcass traits results are presented in Table 2. Slaughter weight and cold carcass weight were affected by both genotype and sex as well as their interactions. Regarding genotype, a higher slaughter weight (by 21.9%; p<0.001) and cold carcass weight (by 24.2%; p<0.001) were detected in NG, and in terms of sex, a higher slaughter weight (by 14.4%; p<0.001) and cold carcass weight (by 16.2%; p<0.001) were observed in males. The effect of genotype was also found in the thigh meat percentage, which was higher (by 5.6%; p = 0.048) in CG compared to NG. The dressing-out percentage and the thigh and abdominal fat percentages were not influenced by any of the studied factors.

### Meat fatty acid composition and lipid metabolism

The dry matter and fat content, fatty acid composition, nutritional indexes and the lipid metabolism indexes of the thigh meat results are shown in Table 3. Dry matter content and fat content were not affected by any of the studied factors.

Genotype affected the contents of some fatty acids and their groups. The palmitoleic (p = 0.047) and eicosenoic (p< 0.001) acid contents were higher in NG, whereas the linoleic (p = 0.002), eicosapentaenic (p = 0.017), total n-6 (p = 0.003) and total polyunsaturated fatty acid (PUFA; p = 0.006) contents were higher in CG. Sex affected fatty acid composition to a lesser extent than genotype; differences between males and females were observed in the linoleic (p<0.001), α-linolenic (p = 0.038), total n-6 (p = 0.016) and total PUFA (p = 0.028) contents, all of which were higher in females.

In terms of nutritional indexes related to human health, the PUFA n-6/PUFA n-3 ratio was affected by both genotype and sex, and a more favourable (p = 0.010) value of this index was detected in NG. With respect to sex, lower PUFA n-6/PUFA n-3 ratio (p = 0.010) values were observed in males. AI, TI, PI, and HH were not influenced by any of the studied factors.

There were almost no differences in lipid metabolism indexes between groups except for the Δ^5^ – Δ^6^ desaturase index (p = 0.006), which was higher in males. None of the evaluated characteristics of the meat dry matter and fat contents, fatty acid composition, nutritional indexes and lipid metabolism indexes were affected by the interaction of genotype and sex.

### Meat oxidative stability

The oxidative stability results are presented in Table 4. Significant effects of genotype, sex and storage time were detected. The level of TBARS in the goose meat increased with storage time (p<0.001). Regarding genotype, the MDA content was lower (by 82.8%; p<0.001) in NG, and in terms of sex, a lower (by 77.2%; p<0.001) TBARS level was recorded in males. An interaction was observed between genotype, sex and storage time (p<0.001). The highest MDA content was found in the meat of CG females after 5 days of storage, whereas the lowest content was in the meat of CG males at 0 days of storage.

## DISCUSSION

The results of the present study show differences between the two genotypes in growth performance and some carcass traits. These results were not surprising as contrasting genotypes (native breed and hybrid) were compared, and it is well known that hybrid geese typically achieve higher growth rates than native breeds. Our results are similar to those of Kapkowska et al [21], who compared fattening in the Polish native goose breed Zatorska and the crossbreed White Kołuda. The only difference between the results of this previous study and the present study is that Kapkowska et al [21] also detected a significantly higher dressing-out percentage in crossbreed compared to the native breed, whereas there were no differences in the dressing percentage between genotypes in the present study, although nonsignificantly higher values were reached in the hybrid goose. Regarding sex, significant differences in growth performance between males and females suggest a degree of sexual dimorphism in geese, which is expressed as a higher growth rate in males. Consistent with our study, Saatci et al [22] and Kapkowska et al [21] observed higher slaughter and carcass weights in males, and similar variations in the carcass traits of Czech goose and a commercial hybrid depending on genotype and sex were detected by Uhlířová et al [23].

The effect of genotype was also reflected in the fatty acid composition of the thigh meat. The major fatty acids in both genotypes were palmitic, oleic and linoleic acids. None of the saturated fatty acids were affected by any of the studied factors in the present study, whereas significant differences between genotypes or sexes were observed in some monounsaturated fatty acids (MUFA) and PUFA and in total n-6 and total PUFA. Regarding individual fatty acids, genotype significantly affected the palmitoleic, eicosenoic, linoleic and eicosapentaenic acid contents. The contents of palmitoleic and eicosenoic acid were higher in NG, whereas the contents of linoleic and eicosapentaenic acids were higher in CG. The effect of sex was detected in the linoleic and α-linolenic acid contents; both were higher in females.The trends observed in the present study were similar to those described in Haraf et al [2], who found that the meat of the pure goose breeds Kartuska and Lubelska contained less MUFA and more PUFA than the intensively reared hybrid White Kołuda. Dal Bosco et al [1] stated that breed was a source of variation in the main fatty acids in chickens, but they attributed differences between slow-growing (pure breeds), medium-growing and fast-growing genotypes to differences in pasture intake or the intramuscular fat content. Adittionaly, Okruszek et al [7] observed different meat fatty acid compositions among various flocks of Polish native goose breeds despite of rearing under the same environmental conditions and feeding regime, and differences in the fat content of the muscle tissue were also detected among the genotypes. De Smet et al [5] reported that general differences in the meat fatty acid profile among various genotypes are often associated with the differences in the fat content of the meat. However, the above explanations seem to be irrelevant to the present experiment, as the fat content of the thigh muscle tissue did not differ between CG and NG, and both genotypes were provided identical feed mixtures. Moreover, the lipid metabolism indexes did not differ between CG and NG, so this was also probably not the source of variation in meat fatty acid composition between CG and NG. De Smet et al [5] suggested that the reason for genotype differences in meat fatty acid composition might be due to differences in gene expression, among other factors. However, further studies should be conducted to clarify this issue. In terms of sex, our finding of higher PUFA contents, including linoleic and α-linolenic acid, in female geese is consistent with results found in pigs, in which higher PUFA concentrations have been repeatedly detected in gilts, even after correcting for differences in fat content [5]. Dal Bosco et al [1] reported that the lower contents of linoleic and α-linolenic acid together with higher Δ^5^ – Δ^6^ desaturase index values in chickens indicate more efficient long-chain acid synthesis, and our study confirmed this relationship. Furthermore, the higher content of α-linolenic acid in females could be explained by the role of steroid hormones [24]. In rats, the n3 long-chain PUFA content of plasma and tissues appeared to be positively related to the concentrations of oestradiol and progesterone and negatively associated with concentrations of testosterone [25].

In terms of nutritional indexes related to human health, effects of both genotype and sex on the PUFA n-6/PUFA n-3 ratio were observed, reflecting both genotype and sex related differences in the total PUFA n-6 content. In the present experiment, this index was higher (9.51 on average) than the recommended values, which should vary from 1.00 to 4.00 [26], and was similar to those observed in various Polish native goose breeds by Okruszek et al [7]. In contrast, the PUFA n-6/PUFA n-3 ratio in Western diets is approximately 15.00 to 16.70 [26].

Differences in oxidative stability with genotype and sex could be related to different PUFA contents; in the present study, the MDA content of the meat increased with greater PUFA content. Castellini et al [27] also observed this relationship in chickens. Furthermore, Castellini et al [28] compared the meat oxidative stability of chickens selected for organic rearing and that of chickens suitable for intensive production systems, and detected lower TBARS levels in chickens intended for organic rearing, although both genotypes were reared under the same (organic) conditions during the study. They explained this finding by the higher intake of grass (antioxidants), leaner meat and more active behaviour (more walking) of the chickens selected for organic rearing. Allesio et al [29] showed that increased locomotory activity is correlated with a more efficient mechanism for controlling muscle oxidative metabolism and free radical production. In the present experiment, the two genotypes were housed in littered pens, but CG is usually reared in free-range conditions and likely could not move sufficiently in the littered pens. Therefore, the MDA content of CG meat could be higher than that of NG meat. However, Skřivanová et al [30] observed better oxidative stability in the breast meat of indoor chickens compared to free-range chickens. Furthermore, storage time affected lipid oxidation in the meat of CG females in this study, which had an extremely high MDA content (13.85 mg/kg) after 5 days of storage. It seems that CG females are more sensitive to caged rearing and the lack of antioxidants that are available in the pasture, which resulted in significantly lower meat oxidative stability after 5 days of storage compared to other groups of geese. On the other hand, the highest MDA content in CG might have been influenced by a higher PUFA content. Further experiments should be performed to elucidate this finding and to increase the information about meat oxidative stability in geese.

## CONCLUSION

The results of the present study showed that CG did not reach growth rates as high as those of the hybrid goose, but the dressing-out percentages of both genotypes were comparable. The advantage of CG is its favourable fatty acid profile characterized by a higher PUFA content, particularly linoleic and eicosapentaenic acids, but its PUFA n-6/PUFA n-3 ratio, which is one of the most important indexes related to human health, was somewhat higher than in NG. The advantage of NG is its relatively high growth rate and higher meat oxidative stability compared to CG. Based on these results, it can be concluded that both CG and NG are able to provide meat of a good quality that could satisfy consumer demand. However, genotype appropriate rearing conditions should be considered; results of the present study suggest that an organic rearing system is more appropriate for CG.

## Figures and Tables

**Table 1:** Ingredients and chemical compositions of the feed mixtures

Items	VH 1	VH 2
Ingredients (%)		
Wheat	36.50	27.45
Maize	23.00	40.00
Soybean meal	29.00	20.00
Meatbone meal	3.50	4.00
Fish meal	2.00	-
Fat	2.00	4.00
MET (40%)	0.30	0.15
LYS (40%)	0.20	-
Vitamin premix	1.00	1.00
Dicalcium phosphate	1.00	1.30
Salt	0.30	0.40
Limestone	1.20	1.70
Chemical composition		
Crude protein (%)	24.35	18.17
Metabolizable energy (MJ/kg)	11.52	12.83

MET, methionine; LYS, lysine.

**Table 2:** Growth performance and carcass traits as affected by genotype and sex

Items	Czech goose		Novohradska goose		RMSE	p value		
		
	Male	Female	Male	Female		Genotype	Sex	Genotype×sex
Growth performance								
Initial body weight (g)	98^d^	105^c^	119^b^	121^a^	0	<0.001	<0.001	<0.001
Final body weight (g)	3,734^c^	3,439^d^	4,962^a^	4,165^b^	282	<0.001	<0.001	<0.001
Daily weight gain (g)	64.9^c^	59.5^d^	86.5^a^	72.2^b^	5.0	<0.001	<0.001	<0.001
Daily feed intake (g)	192^c^	159^d^	211^a^	204^b^	0	<0.001	<0.001	<0.001
Feed conversion	3.0^a^	2.7^b^	2.5^c^	2.9^a^	0.2	<0.001	0.215	<0.001
Carcass traits								
Slaughter weight (g)	3,830^c^	3,525^d^	5,026^a^	4,138^b^	130	<0.001	<0.001	<0.001
Cold carcass weight (g)	2,358^c^	2,105^d^	3,130^a^	2,560^b^	103	<0.001	<0.001	<0.001
Dressing-out percentage (%)	68.2	67.3	68.3	68.7	1.7	0.210	0.678	0.293
Thighs percentage (%)	24.0	25.3	23.5	23.4	1.9	0.084	0.380	0.303
Thigh meat percentage (%)	15.5	15.6	14.9	14.5	1.1	0.048	0.774	0.473
Abdominal fat percentage (%)	2.8	2.8	3.3	3.2	0.9	0.175	0.843	0.888

RMSE, root mean square error.

a,b,c,d Means with different letters within the same row differ at p<0.05.

**Table 3:** Chemical composition, fatty acid composition, nutritional indexes and lipid metabolism indexes of goose meat according to genotype and sex

Items	Czech goose		Novohradska goose		RMSE	p value		
		
	Male	Female	Male	Female		Genotype	Sex	Genotype×sex
Chemical composition (%)								
Dry matter	24.18	24.42	23.95	24.05	0.52	0.115	0.374	0.702
Fat	2.02	2.43	2.18	2.25	0.57	0.966	0.248	0.407
Fatty acid (%)								
Myristic	0.86	0.71	0.73	0.73	0.19	0.410	0.270	0.255
Palmitic	19.84	19.25	20.08	19.88	0.90	0.184	0.227	0.539
Stearic	10.63	9.61	10.51	10.06	1.02	0.652	0.051	0.441
Palmitoleic	2.53	2.46	2.66	2.73	0.26	0.047	0.994	0.446
Oleic	37.17	37.29	38.88	38.15	2.40	0.141	0.718	0.619
Eicosenoic	0.26	0.26	0.30	0.29	0.03	<0.001	0.616	0.738
Linoleic	16.71	19.30	15.46	16.84	1.52	0.002	<0.001	0.270
Arachidonic	5.50	4.99	4.97	4.96	0.83	0.344	0.379	0.411
α-linolenic	0.99	1.13	0.88	1.05	0.20	0.196	0.038	0.826
Eicosapentaenic	0.03	0.03	0.02	0.03	0.01	0.017	0.619	0.066
Clupanodonic	0.48	0.44	0.48	0.48	0.07	0.408	0.343	0.543
Docosahexaenic	0.93	0.80	0.92	0.82	0.16	0.869	0.057	0.791
Total SFA	32.13	30.28	32.01	31.41	1.73	0.414	0.054	0.314
Total MUFA	42.33	42.22	44.34	43.55	2.49	0.068	0.608	0.702
Total n-6	22.99	24.99	21.21	22.53	1.83	0.003	0.016	0.606
Total n-3	2.47	2.44	2.35	2.42	0.28	0.524	0.867	0.595
Total PUFA	25.54	27.51	23.65	25.04	2.05	0.006	0.028	0.695
Nutritional indexes								
PUFA n-6/PUFA n-3 ratio	9.36	10.28	9.04	9.36	0.64	0.010	0.010	0.205
Atherogenicity index	0.35	0.32	0.34	0.33	0.03	0.610	0.130	0.325
Thrombogenicity index	0.78	0.72	0.78	0.76	0.06	0.329	0.055	0.408
Proxidability index	53.84	53.19	50.21	50.96	5.33	0.131	0.977	0.713
hypocholesterolemic/hypercholesterolemic ratio	3.00	3.21	2.97	3.04	0.23	0.210	0.091	0.368
Lipid metabolism indexes								
Elongase	0.54	0.50	0.52	0.51	0.05	0.941	0.178	0.571
Thioesterase	24.55	27.79	29.18	27.37	4.85	0.229	0.682	0.153
Δ^9^ desaturase (16)	11.32	11.35	11.69	12.05	1.01	0.145	0.590	0.639
Δ^9^ desaturase (18)	77.67	79.45	78.68	79.11	2.54	0.711	0.229	0.457
Δ^9^ desaturase (16+18)	56.71	57.92	57.67	58.77	2.40	0.292	0.183	0.951
Δ^5^ – Δ^6^ desaturase	29.31	24.36	29.28	27.01	3.45	0.290	0.006	0.282

RMSE, root mean square error; SFA, saturated fatty acids; MUFA, monounsaturated fatty acids; PUFA, polyunsaturated fatty acids.

**Table 4:** Oxidative stability of goose meat as affected by genotype, sex, and storage time

Items	Czech goose		Novohradska goose		RMSE	p value			
		
	Male	Female	Male	Female		Genotype	Sex	Storage time	G^2^×S^3^×ST^4^
TBARS (mg MDA/kg)									
0 days of storage	0.49^b^	0.94^b^	0.55^b^	0.74^b^	2.26	<0.001	<0.001	<0.001	<0.001
3 days of storage	1.45^b^	3.42^b^	0.95^b^	2.14^b^	-	-	-	-	-
5 days of storage	4.60^b^	13.85^a^	2.71^b^	3.17^b^	-	-	-	-	-

RMSE, root mean square error; G, genotype; S, sex; ST, storage time; TBARS, thiobarbituric acid reactive substances; MDA, malondialdehyde.

a,b Means with different letters within the same row differ at p<0.05.
